# Characteristics of Invasive Pulmonary Fungal Diseases Diagnosed by Pathological Examination

**DOI:** 10.1155/2021/5944518

**Published:** 2021-10-28

**Authors:** Dong Zhang, Xin Li, Junquan Zhang, Junping Wu, Xin Sun

**Affiliations:** ^1^Department of Tuberculosis, Tianjin Haihe Hospital, Tianjin University, Tianjin, China; ^2^Tianjin Key Laboratory of Lung Regenerative Medicine, Tianjin Haihe Hospital, Tianjin University, Tianjin, China; ^3^Department of Pathology, Tianjin Haihe Hospital, Tianjin University, Tianjin, China; ^4^Department of Medical Records, Tianjin Haihe Hospital, Tianjin University, Tianjin, China; ^5^Department of Respiratory Medicine, Tianjin Haihe Hospital, Tianjin University, Tianjin, China

## Abstract

**Objective:**

To explore the characteristics of invasive pulmonary fungal disease and the spectrum of pathogens causing invasive pulmonary fungal disease diagnosed by pathological examination using fungal stains.

**Methods:**

Patients with an invasive pulmonary fungal disease diagnosed by histopathological analysis through the use of fungal stains (including Grocott's methenamine silver and periodic acid-Schiff stains) were included in this study. The clinical records, radiological reports, pathology, and fungal culture results were reviewed.

**Results:**

Forty-eight invasive pulmonary fungal disease patients diagnosed by histopathological analysis in the Tianjin Haihe Hospital (including 8 cases obtained by pulmonary resection, 35 cases by fiberoptic bronchoscopic biopsy, and 5 cases by percutaneous lung biopsy) were included. There were 24 male and 24 female patients, aged 21–80 years (53 ± 13 years). There were 37 cases of pulmonary aspergillosis, 4 cases of pulmonary cryptococcosis, 2 cases of pulmonary mucormycosis, and 5 in which pathogens were not determined due to limited tissue availability. Among 48 cases, 32 specimens were submitted to fungal culture. No fungus was detected in culture, although 26 cases of fungus infections were diagnosed by histopathological analysis. Only 3 cases were consistent between histopathological and culture results. In 3 cases, the pathogen was identified as *Aspergillus* spp. by the histopathological analysis, while the contrasting fungal culture results identified *Candida albicans*.

**Conclusion:**

*Candida albicans* pneumonia was rare, while aspergillosis was common in invasive pulmonary fungal disease diagnosed by histopathological analysis. The majority of patients with an invasive pulmonary fungal disease were culture-negative. Although culture can clarify the fungal pathogen species, it has low sensitivity. Pathological examination with fungal stains has its advantages in diagnosing fungal disease; therefore, more attention should be paid to the role of pathological examination in the diagnosis of fungal disease.

## 1. Introduction

The incidence of invasive pulmonary fungal disease is increasing in China with the wide use of immunosuppressants and antibiotics. Individuals with underlying HIV/AIDS, malignant tumors, oncohematological disorders, transplantation, tuberculosis, chronic respiratory diseases, and diabetes are at a high risk of developing a fungal infection [1, 2]. Invasive pulmonary fungal disease, especially invasive mucormycosis, has been reported frequently in India with the recent emergence of the coronavirus disease (COVID-19) disease. The patients treated with corticosteroids and broad-spectrum antibiotics for controlling COVID-19 are at high risk of mucormycosis. However, it is challenging to diagnose and treat fungal infections, since only approximately 50% of patients are diagnosed before death [3, 4].

Fungal culture is the diagnostic golden standard for invasive pulmonary fungal infection. It can identify the fungal pathogen species and test for antifungal susceptibility, but its low sensitivity often delays the diagnosis [5, 6]. Nonculture methods, such as PCR-based assays and serologic tests, should be considered in the diagnosis of invasive fungal infections, although the effect of antifungal therapy decreases the yield of these tests. Histopathological examination with fungal stains, which is a cost-effective nonculture method, can provide another method to diagnose fungal infections. Microscopic examination can help diagnose infections through the identification of morphological features of fungi. Typical hyphae, pseudohyphae, and budding yeast cells can be identified by histochemical fungal stains because some dyes can selectively bind to the fungal cell wall, thus helping to identify the fungal structures [7, 8].

Previous research often focused on the role of laboratory tests and culture in invasive pulmonary fungal disease. Data of histopathological findings with fungal stains in invasive pulmonary fungal disease are scarce. This study compared traditional culture and histopathological methods to improve the diagnosis of pulmonary fungal infection.

## 2. Methods

Forty-eight patients with invasive pulmonary fungal diseases diagnosed by pathology and admitted to the Tianjin Haihe Hospital from 2012 to 2020 were included. The clinical records, radiology reports, serological test results, pathology reports, and culture results were reviewed. Hematoxylin and eosin- (H&E-) stained microscopic slides and slides with fungal stains (Grocott's methenamine silver (GMS) and periodic acid-Schiff (PAS)) were analyzed. This study was approved by the Tianjin Haihe Hospital' Ethics Review Board.

### 2.1. Statistical Analysis

Descriptive statistical analyzes were performed using Microsoft Excel 2013.

## 3. Results

### 3.1. Clinical Manifestations

There were 24 male and 24 female patients. The patients ranged in age from 21 to 80 years (53 ± 13 years). Risk factors for invasive pulmonary fungal disease included diabetes (15/48, 31.25%), tuberculosis (12/48, 25%), bronchiectasis (9/48, 18.75%), lung carcinoma (6/48, 12.5%), coronary heart disease (5/48, 10.42%), chronic obstructive pulmonary disease (2/48, 4.17%), cerebral vascular disease (1/48, 2.08%), and acute myeloid leukemia (1/48, 2.08%). Among 48 patients, 28 (70%) were smokers and ex-smokers (1.29 ± 0.53 packs/day, period: 27.41 ± 10.79 years), and 20 (30%) were nonsmokers.

Symptoms included cough in 37 patients (77.08%), dyspnea in 8 patients (16.67%), fever in 19 patients (39.58%), hemoptysis in 18 patients (37.5%), and chest pain in 2 patients (4.17%).

Among the 48 specimens, 35 were transbronchial biopsies, 8 were surgical specimens, and 5 were percutaneous lung biopsies.

### 3.2. Spectrum of Pathogens and Culture Results

GMS and PAS stainings were performed. A total of 43 of 48 cases had definitive pathology reports. Among the 48 cases, the pathogens included *Aspergillus* spp. (37/48, 77.08%), *Cryptococcus neoformans* (4/48, 8.33%), and *Mucorales* (2/48, 4.17%), while 5 cases (10.42%) were left without a diagnosis due to lack of available tissue. Pathogen cultures were performed on 32 of 48 cases. Among specimens submitted to culture, 27 were sputum. The results of fungal cultures were negative in 26 cases. Only 6 cases were positive, of which 3 patients had the same results between pathology and culture and the other 3 were diagnosed with culture-positive for *Candida albicans*, despite pathology reports identifying *Aspergillus* spp. Conventional biomarkers galactomannan (GM) and 1,3-*β*-d-glucan (BDG) were positive, while 1,3-*β*-d-glucan (BDG) was negative in these 3 contrasting cases (Table 1).

## 4. Discussion

In this study, we identified several species that caused invasive pulmonary fungal disease diagnosed by pathology, namely, *Aspergillus* spp., *Cryptococcus neoformans*, and *Mucorales*. There were some differences in the morbidity of different fungal species between our study and those of other reports. In a large series of autopsy reports, data of fungal diseases were reviewed and investigated by collecting studies published in PubMed from 2008 to 2013 [3]. This study identified the following species of fungi: *Aspergillus* spp., *Candida albicans*, *Cryptococcus neoformans*, and *Mucorales*. In a Chinese report, Liu et al. [9] have shown the incidence of different pathogens causing invasive pulmonary fungal disease: *Aspergillus* spp. 39%, *Candida albicans* 34%, *Cryptococcus neoformans* 16%, and *Mucorales* 2%. These incidences are different from our results. One possible explanation is that these authors analyzed patients diagnosed by clinical presentation, culture, laboratory tests, and pathology results. Our study only included patients diagnosed by histopathological analyzes. The difference between our study and that of Pappas et al. is mainly on the incidence of *Candida albicans*. *Candida* pneumonia can only be diagnosed in blood and other types of specimen collected under a sterile environment. It also requires histological evidence of invasive disease [10, 11]. The reason of a low incidence of *Candida albicans* pneumonia diagnosed by pathological examination may be due to a relatively short course of the disease. The likelihood of performing an endoscopy is low; therefore, biopsy tissues of *Candida* pneumonia are not common in pathology.

To investigate the role of fungal stains in diagnosing pulmonary fungal disease, we compared the results between culture and histopathological examination with special fungal stains. In the 48 cases diagnosed by pathology, 32 samples were submitted to fungal culture. There was a low consistency in detecting fungal pathogens between pathological examination and the traditional culture method. Interestingly, we had 26 culture-negative cases, in spite of a positive diagnosis by histopathological examination. This high ratio of culture-negative cases suggests that the sensitivity of fungal culture is low, and a negative fungal culture does not rule out an invasive fungal infection. If physicians depend solely on the results of fungal cultures, this may delay the optimal treatment time and harm patient recovery.

Interestingly, only 3 patients had the same results in both pathology and culture. Another 3 cases had contrasting results in which the pathogen found in pathology was *Aspergillus* spp., although the culture result indicated *Candida albicans*. The serological test results for *Candida* mannan antigen, antibody, and 1,3-*β*-d-glucan (BDG) were negative, while that of galactomannan (GM) was positive. *Candida* organisms can colonize the mouth and skin [12, 13]. The culture samples were sputum obtained from nonsterile sites, which could be contaminated with oral pathogens, suggesting that, in these patients, *Candida albicans* could be part of the microbiota and not the causative pathogen of the infection. According to serological test results and pathological examination, the patients were treated as aspergillosis, and the prognosis was favorable.

The most common symptoms of an invasive pulmonary fungal disease were cough and fever in this study. Most patients had underlying diseases, such as diabetes, tuberculosis, chronic pulmonary diseases, and malignant tumors. Nodular consolidation, cavity, patches, and stripe shadows were common signs in CT results. As the symptoms and image findings were unclear, most patients were not considered to have an invasive pulmonary fungal disease at first; therefore, the rate of tissue samples submitted to culture was low. Clinicians should pay attention to this phenomenon and increase the rate of tissue biopsy submitted to fungal culture.

There are some limitations to this study. First, the rate of sputum specimens was high (27/32, 84.38%) in fungal culture. In contrast, tissues were not submitted for culture as physicians performed the biopsy thinking of a neoplasm. As invasive pulmonary fungal disease was not common, the number of patients included in this study was low. PCR-based assays were not performed in this study. It is possible to identify species rapidly from clinical samples by using molecular tools now.

In conclusion, *Candida*-induced pneumonia was rare, and aspergillosis was common in invasive pulmonary fungal disease diagnosed by pathology. The sensitivity rate of traditional culture methods was lower than that of pathology in diagnosing fungal diseases. Clinicians and pathologists should be aware of histological findings in tissue specimens. In regular practice, special fungal stains should be used for surgical specimens in which there is a suspicion of fungal diseases. Pathology is a useful method to help reduce the time to diagnosis of invasive pulmonary fungal disease, especially for certain fungi whose morphological features can be identified by microscopic examination. A combination of pathology, culture, serology, and PCR-based molecular examination is the best approach to efficiently diagnose invasive pulmonary fungal infection.

## Figures and Tables

**Table 1:** Spectrum of pathogens causing invasive pulmonary fungal disease diagnosed by pathology.

	Pathology	Culture
*Aspergillus* spp.	37	2
*Cryptococcus neoformans*	4	0
*Mucorales*	2	1
*Candida albicans*	0	3
Pathogens were not determined	5	0
No pathogen	0	26
Total	48	32

## Data Availability

The datasets used and analyzed during this study are available from the corresponding author upon request.
